# Pharmacometabolomics of Response to Sertraline and to Placebo in Major Depressive Disorder – Possible Role for Methoxyindole Pathway

**DOI:** 10.1371/journal.pone.0068283

**Published:** 2013-07-17

**Authors:** Hongjie Zhu, Mikhail B. Bogdanov, Stephen H. Boyle, Wayne Matson, Swati Sharma, Samantha Matson, Erik Churchill, Oliver Fiehn, John A. Rush, Ranga R. Krishnan, Eve Pickering, Marielle Delnomdedieu, Rima Kaddurah-Daouk, Pharmacometabolomics Research Network

**Affiliations:** 1 Department of Psychiatry and Behavioral Sciences, Duke University, Durham, North Carolina, United States of America; 2 Department of Neurology and Neuroscience Weill Cornell Medical College, New York, New York, United States of America; 3 Department of Systems Biochemistry, Bedford VA Medical Center, Bedford, Massachusetts, United States of America; 4 Massachusetts General Hospital, Boston, Massachusetts, United States of America; 5 Genome Center, University of California Davis, Davis, California, United States of America; 6 Duke-NUS Graduate Medical School, Singapore, Singapore; 7 Pfizer Global R&D, Clinical Research Statistics, Groton, Connecticut, United States of America; 8 Pfizer Global R&D, Neuroscience Clinical Research, Groton, Connecticut, United States of America; Imperial College London, United Kingdom

## Abstract

Therapeutic response to selective serotonin (5-HT) reuptake inhibitors in Major Depressive Disorder (MDD) varies considerably among patients, and the onset of antidepressant therapeutic action is delayed until after 2 to 4 weeks of treatment. The objective of this study was to analyze changes within methoxyindole and kynurenine (KYN) branches of tryptophan pathway to determine whether differential regulation within these branches may contribute to mechanism of variation in response to treatment. Metabolomics approach was used to characterize early biochemical changes in tryptophan pathway and correlated biochemical changes with treatment outcome. Outpatients with MDD were randomly assigned to sertraline (n = 35) or placebo (n = 40) in a double-blind 4-week trial; response to treatment was measured using the 17-item Hamilton Rating Scale for Depression (HAMD_17_). Targeted electrochemistry based metabolomic platform (LCECA) was used to profile serum samples from MDD patients. The response rate was slightly higher for sertraline than for placebo (21/35 [60%] vs. 20/40 [50%], respectively, χ^2^(1)  = 0.75, p = 0.39). Patients showing a good response to sertraline had higher pretreatment levels of 5-methoxytryptamine (5-MTPM), greater reduction in 5-MTPM levels after treatment, an increase in 5-Methoxytryptophol (5-MTPOL) and Melatonin (MEL) levels, and decreases in the (KYN)/MEL and 3-Hydroxykynurenine (3-OHKY)/MEL ratios post-treatment compared to pretreatment. These changes were not seen in the patients showing poor response to sertraline. In the placebo group, more favorable treatment outcome was associated with increases in 5-MTPOL and MEL levels and significant decreases in the KYN/MEL and 3-OHKY/MEL; changes in 5-MTPM levels were not associated with the 4-week response. These results suggest that recovery from a depressed state due to treatment with drug or with placebo could be associated with preferential utilization of serotonin for production of melatonin and 5-MTPOL.

## Introduction

Selective serotonin (5-HT) reuptake inhibitors (SSRIs) are the most commonly prescribed antidepressant medications for the treatment of Major Depressive Disorder (MDD). The primary target for SSRIs is the 5-HT transporter, inhibition of the transporter initiates multiple changes within different branches of the tryptophan pathway; yet antidepressant effects are very complex, and several pathways have been implicated in their mechanism of action, in addition to affecting serotoninergic neurotransmission, SSRIs also influence other systems, such as dopamine [1]–[3] and norepinephrine [4]–[6], as well as glutamate [7]–[9] and γ-aminobutyric acid [10]–[12].

Response to current therapies in treating MDD varies considerably, and onset of antidepressant therapeutic action typically does not occur until after 2 to 4 weeks of treatment which delays clinicians from knowing whether an antidepressant is going to work for a particular patient [13],[14]. Mechanisms underlying the variation in the likelihood and timing of treatment response in depression remain largely unknown, different mechanisms are likely involved due to the multiple effects of antidepressants and the heterogeneity of the disease [15]–[22]. The placebo effect adds even more complexity; the mechanisms underlying the placebo response may differ from those involved in the response to SSRIs, thus making it more difficult to identify mechanisms involved in the therapeutic action of the medication [23]–[25]. Placebo response could also reflect spontaneous improvement and thus change in placebo group that corresponds to improvement in depression could reflect disease state alterations independent of medication.

Metabolomics tools enable identification and quantification of hundreds to thousands of compounds that can report on changes in biochemical pathways [26]. Recently, we started to apply metabolomics approaches to map global biochemical changes in depression and pathways implicated in mechanism of variation of response to SSRIs [27]–[29]. Recently we found that metabolic profiles in MDD patients prior to treatment could distinguish responders from non-responders to sertraline or placebo; several tryptophan pathway metabolites contributed to the separation of responders and non-responders in that study [30]. In the present study we provide detailed mapping of effects of sertraline and placebo on key neurotransmitter pathways after one week and four weeks of treatment and correlate biochemical changes with treatment outcomes. A detailed analysis of changes within methoxyindole and kynurenine (KYN) branches of tryptophan pathway is conducted based on findings that differential regulation within these branches may contribute to mechanism of variation in response to treatment. The metabotype of a depressed patient at baseline and the biochemical changes induced by treatment can inform about treatment outcomes and can highlight heterogeneity in a complex disease such as depression.

## Materials and Methods

### Study Design

The 75 patients in this report were a subset of the 165 patients who entered a randomized, double-blind, flexible dosing, placebo-controlled study performed at 12 clinical sites. Sertraline dosing was started at 50 mg/day at baseline (week 0), with dose increased up to 100 mg/day at week 1 and up to 150 mg/day at week 2, as seen needed by the treating clinician. Subjects selected for this study were those with serum samples and HAMD_17_ scores available at baseline and 1 week and at 4 weeks (+/−1 week) after treatment. Finally, the subset of participants in this study largely overlaps with those used in our previous study [30].

### Patients and Samples

Study participants were outpatients, 18–65 years of age, from various clinical sites across the United States. Patients had a primary diagnosis of nonpsychotic MDD by the criteria specified in the Diagnostic and Statistical Manual of Mental Disorders, 4th Edition, with symptoms of depression present for at least one month prior to screening, and a total baseline score >22 on the 17-item HAMD_17_ at screening. A complete description of the inclusion and exclusion used in this study can be found in Table S1. The study protocol was developed in accordance with the principles of the Declaration of Helsinki. All patients provided written informed consent. The study was sponsored and monitored by Pfizer, Inc. Each site's institutional review board approved and oversaw the study. The primary outcome measure was the HAMD_17_, used to assess depressive symptom severity. Measures were gathered at baseline, and weeks 1 and 4 of treatment at the patient's visits. Serum samples were collected using standard protocol at baseline, week 1 and week 4 of treatment at the clinic visits. Whole blood was collected in serum collection tubes and inverted 5–8 times. Serum samples were allowed to clot at room temperature for 60 minutes and then centrifuged at 1,300×g for 15 minutes. Following centrifugation supernatant was frozen at −80°C until analysis.

### Metabolomic Profiling

Samples were analyzed using a liquid chromatography electrochemical array (LCECA) platform that has been extensively validated and used in our prior studies in neurodegenerative and psychiatric disorders [30]–[34]. The method includes multiple compounds from the tyrosine, tryptophan, sulfur amino acids and purine pathways, and markers of oxidative stress and protection (for the list of metabolites and abbreviations see Table 1). Levels of 5-Methoxytryptamine (5-MTPM) were measured using a gas chromatography time-of-flight-mass spectrometry (GCTOF-MS) platform [29]. Samples were prepared for analysis as previously described [30]. During the sample preparation, pools were created from equal volumes of subaliquots of all samples. Concentrations of metabolites in the individual samples were expressed as a percentage of the concentration of those metabolites in the averaged pool.

**Table 1:** Metabolites Quantified by the LCECA platform.

Pathway	Metabolite	Abbreviation
Tryptophan	3-Hydroxykynurenine	3-OHKY
	5-Hydroxyindoleacetic Acid	5-HIAA
	5-Hydroxytryptophan	5-HTP
	*N*-Acetylserotonin	NA-5-HT
	Anthranillic Acid	ANA
	Indole-3-lactic Acid	I-3-LA
	Melatonin	MEL
	Serotonin	5-HT
	Tryptophan	TRP
	5-Methoxytryptophol	5-MTPOL
	Tryptophol	TRPOL
	Kynurenine	KYN
Tyrosine	3-*O*-methyldopa	3-OMD
	4-Hydroxyphenylacetic Acid	4-HPAC
	3,4-Dihydroxyphenylacetic Acid	DOPAC
	3,4-Dihydroxymandelic Acid	DIOHMAL
	Homogentistic Acid	HGA
	Homovanillic Acid	HVA
	L-DOPA	LD
	Methoxyhydroxyphenlyglycol	MHPG
	Tyrosine	TYR
	Vanillylmandelic Acid	VMA
Phenylalanine	4-Hydroxybenzoic Acid	4-HBAC
	4-Hydroxyphenyllactic Acid	4-HPLA
Purine	7-Methylxanthine	7-MXAN
	Guanosine	GR
	Guanosine Mono Phosphate	GRMP
	Hypoxanthine	HX
	Uric Acid	UA
	Xanthine	XAN
	Xanthosine	XANTH
Cysteine, Glutathione	Glutathione (reduced)	GSH
	Cysteine	CYS
Antioxidants	Delta-Tocopherol	DTOCO
	Alpha-Tocopherol	ATOCO
One Carbon Metabolism	Methionine	METH
Other	Vanillic Acid	VANA

### Statistical Methods

Data preprocessing: We first removed the metabolites that have more than 40% missing values. Data from one subject who had more than 50% metabolite data missing was excluded from analysis. Most metabolites have right-skewed distribution; log transformation was thus applied to the metabolite concentrations at all three time points to induce normality of the data. Principal components analysis (PCA; SIMCA-P+12.0) was used to identify outliers. The analysis was performed for each time point. All metabolites were first standardized to have mean 0 and standard deviation 1. Subjects with ≥3 standard deviations away from the center of data cloud within two principal components (PC's) scores scatter plot were considered outliers and were removed from further analysis.

#### Metabolic signatures of sertraline and placebo

Paired t-tests were used to examine which metabolites changed significantly from baseline to week 1 and from baseline to week 4. These analyses were conducted separately for the sertraline and placebo groups. Between groups t-tests were used to compare baseline to one week and baseline to four week change scores between the sertraline and placebo groups.

#### Correlation analysis

Pearson correlation analysis was used to examine associations of changes in metabolite levels with concurrent change in depressive symptoms. Analyses were conducted separately for one and four week change. Correlations of the sertraline group and placebo group were compared using the two-sample *Z*-test after hyperbolic tangent transformation.

#### Metabolic signatures in good and poor responders to sertraline and placebo

Signatures of response to sertraline and placebo were identified by using paired t-tests to compare week 4 metabolite levels to baseline metabolite levels in the responders and non-responders to therapy. Participants were considered responders if they showed a ≥50% percent reduction in HAMD_17_ scores after four weeks of treatment. These analyses were also performed to evaluate changes in ratios of individual metabolites within (e.g., 5-HT/5-HTP) and between branches of tryptophan pathway (e.g., KYN/MEL).

#### Correlation analysis of tryptophan pathway metabolites

Pearson correlation analysis was used to examine associations of the pre-treatment levels of tryptophan pathway metabolites and ratios to the percent change in HAMD_17_ scores after four weeks of treatment.

For all the above systematic univariate tests, q-values [35] were calculated for controlling multiple testing false discovery rate.

## Results

### Sample Characteristics

The sertraline and placebo groups did not differ significantly in age (44±11.3 years vs. 40±12.9 years, p = 0.15), gender (24/35 [69%] vs. 28/40 [70%] female, p = 0.89) or race (26/35 [74%] vs. 31/40 [78%] white, p = 0.75). The response rate was slightly higher for sertraline than for placebo, but this difference was not statistically significant (21/35 [60%] vs. 20/40 [50%] responders, χ^2^(1) = 0.75, p = 0.39). Age, gender, or race were not associated with percent change in HAMD_17_ scores in either sertraline or placebo group (Table S2). Due to potential diurnal variation in levels of methoxyindoles we also analyzed whether there are differences in blood collection times between sertraline and placebo groups for both responders and non-responders. No significant differences were found (data not shown).

### Metabolic Signatures of Sertraline and Placebo – One Week and Four Weeks Exposure

#### Metabolic signatures of sertraline after one and four weeks of treatment

**Figure 1:**
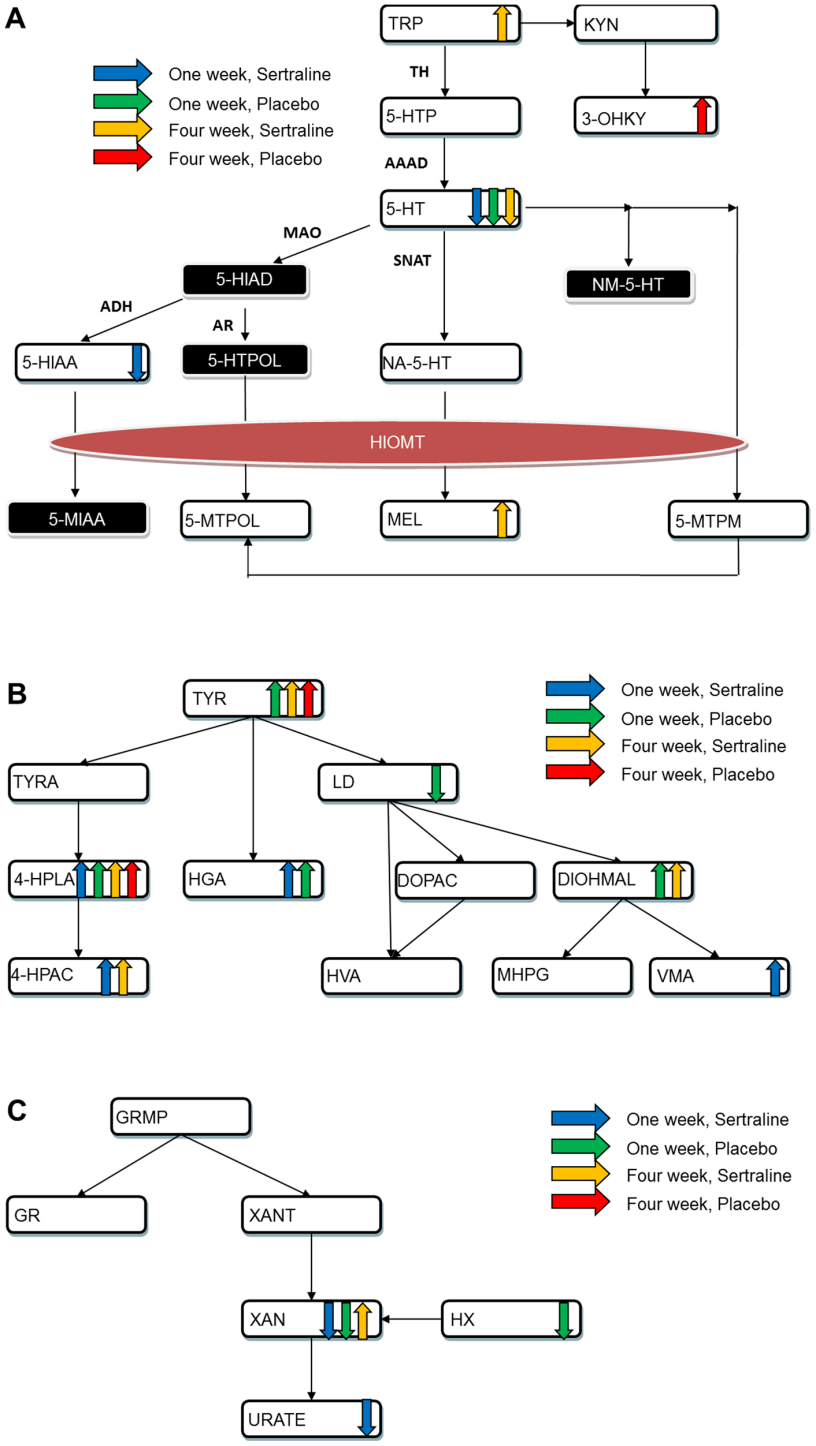
Changes in metabolites within (A) tryptophan, (B) tyrosine and (C) purine metabolism pathways. Abbreviations: HIOMT  =  hydroxyindole-*O*-methyltransferase; 5-HIAD  = 5-Hydroxyindole acetyldehyde; NM-5-HT  =  N-methyl serotonin; 5-HTPOL  = 5-Hydroxytryptophol; 5-MIAA  = 5-Methoxyindole acetic acid; 5-MTPM  = 5-methoxytryptamine. For the remaining metabolites, see Table 1.

Several metabolites changed significantly from baseline to week one in the group receiving sertraline (Table 2). The most significant changes were decreased levels of 5-HT and its major metabolite 5-HIAA. Other metabolites significantly changed following the first week of treatment with sertraline were from the purine and tyrosine/phenylalanine pathways (Table 2). Several metabolites also showed significant changes after four weeks of sertraline administration (Table 3). Similar to one week, the most prominent change in this group was the decrease in 5-HT levels. A number of other metabolites showed significant increases including 4-HPLA, DIOHMAL, tocopherols, 4-HPAC, METH, HGA, TYR, TRP, and MEL. Compounds that changed after one week and four weeks of treatment with sertraline are shown within their respective pathways in Figure 1 A-C.

**Table 2:** Metabolic changes after one week treatment with sertraline and placebo.

Compound	Pathway	Sertraline			Placebo			Comparison	
		Change	P-value	Q-value	Change	P-value	Q-value	P-value	Q-value
TRPOL	Tryptophan	0.033	0.74	0.64	**−0.21**	**0.016**	**0.062**	0.062	0.8
5-HT	Tryptophan	**−0.75**	**2.8E-12**	**2.4E-10**	**−0.21**	**0.014**	**0.062**	**4.7E-06**	**5.5E-04**
5-HIAA	Tryptophan	**−0.17**	**0.00041**	**0.011**	−0.036	0.40	0.45	0.031	0.67
5-HTP	Tryptophan	−0.11	0.21	0.34	−0.019	0.84	0.63	0.49	0.91
5-MTPOL	Tryptophan	0.041	0.50	0.52	0.016	0.81	0.62	0.78	0.94
5-MTPM	Tryptophan	−1.8	0.075	0.22	**−2.2**	**0.031**	**0.090**	0.31	0.91
KYN	Tryptophan	−0.045	0.23	0.35	0.043	0.21	0.29	0.084	0.80
NA-5-HT	Tryptophan	0.0079	0.90	0.71	0.06	0.35	0.41	0.56	0.91
TRP	Tryptophan	0.0036	0.92	0.71	0.063	0.067	0.14	0.23	0.86
3-OHKY	Tryptophan	−0.074	0.20	0.34	0.096	0.087	0.16	0.035	0.67
MEL	Tryptophan	0.34	0.10	0.23	0.3	0.22	0.30	0.89	0.97
LD	Tyrosine	0.081	0.37	0.43	−0.22	0.043	0.11	0.032	0.67
HVA	Tyrosine	0.053	0.46	0.48	0.019	0.8	0.62	0.74	0.94
VMA	Tyrosine	0.11	0.029	0.13	0.041	0.41	0.45	0.31	0.91
DOPAC	Tyrosine	−0.13	0.22	0.34	0.051	0.67	0.59	0.26	0.86
MHPG	Tyrosine	−0.17	0.33	0.42	0.051	0.82	0.62	0.43	0.91
TYR	Tyrosine	0.083	0.10	0.23	**0.11**	**0.024**	**0.073**	0.68	0.92
3-OMD	Tyrosine	−0.12	0.16	0.30	0.14	0.17	0.26	0.05	0.73
4-HPAC	Tyrosine	0.29	0.0044	0.057	0.15	0.072	0.15	0.26	0.86
DIOHMAL	Tyrosine	0.18	0.091	0.23	**0.25**	**0.0057**	**0.032**	0.59	0.91
HGA	Tyrosine	**0.28**	**0.015**	**0.10**	**0.27**	**2.5E-05**	**0.0019**	0.93	0.97
XAN	Purine	−0.19	0.029	0.13	**−0.25**	**0.00017**	**0.0045**	0.56	0.91
HX	Purine	−0.07	0.38	0.44	**−0.22**	**0.0043**	**0.032**	0.17	0.86
GR	Purine	−0.23	0.31	0.40	−0.13	0.56	0.56	0.76	0.94
7-MXAN	Purine	−0.099	0.74	0.64	−0.1	0.72	0.59	0.99	0.98
GRMP	Purine	−0.011	0.90	0.71	−0.033	0.69	0.59	0.85	0.97
XANTH	Purine	0.082	0.16	0.30	−0.022	0.62	0.59	0.15	0.86
UA	Purine	**−0.077**	**0.0061**	**0.057**	0.011	0.71	0.59	0.032	0.67
METH	One Carbon Metabolism	0.097	0.084	0.23	0.14	0.056	0.13	0.64	0.91
4-HPLA	Phenylalanine	**0.1**	**0.0049**	**0.057**	**0.095**	**0.0044**	**0.032**	0.83	0.97
4-HBAC	Phenylalanine	−0.085	0.77	0.65	0.19	0.46	0.49	0.48	0.91
ATOCO	Antioxidant	0.21	0.29	0.40	**0.48**	**0.0048**	**0.032**	0.27	0.86
DTOCO	Antioxidant	0.3	0.30	0.40	**0.5**	**0.039**	**0.10**	0.60	0.91
CYS	Cysteine, Glutathione	−0.034	0.21	0.34	0.039	0.25	0.32	0.089	0.8
GSH	Cysteine, Glutathione	−0.013	0.44	0.47	−0.0067	0.72	0.59	0.81	0.96

Changes in absolute concentrations of metabolites (log-transformed) after one week are shown; positive values – up-regulated metabolites, negative values– down-regulated metabolites. Significant changes are shown in bold. See methods section for the details of statistical analysis. Abbreviations: 5-MTPM – 5-methoxytryptamine; for the remaining metabolites, see Table 1.

**Table 3:** Metabolic changes after four week treatment with sertraline and placebo.

Compound	Pathway	Sertraline			Placebo			Comparison	
		Change	P-value	Q-value	Change	P-value	Q-value	P-value	Q-value
TRPOL	Tryptophan	0.061	0.5	0.49	−0.015	0.88	0.46	0.57	0.92
5-HT	Tryptophan	**−1.9**	**8.70E-12**	**7.10E-10**	−0.018	0.78	0.45	**5.40E-12**	**6.40E-10**
5-HIAA	Tryptophan	−0.06	0.31	0.36	0.015	0.79	0.45	0.36	0.91
5-HTP	Tryptophan	0.12	0.1	0.16	0.0022	0.98	0.5	0.28	0.91
5-MTPOL	Tryptophan	0.13	0.089	0.14	0.092	0.19	0.21	0.68	0.99
5-MTPM	Tryptophan	**−0.29**	**0.0089**	**0.026**	−0.058	0.43	0.36	0.09	0.9
KYN	Tryptophan	0.045	0.16	0.22	0.04	0.26	0.26	0.92	1
NA-5-HT	Tryptophan	0.041	0.45	0.45	0.019	0.74	0.45	0.78	1
TRP	Tryptophan	**0.07**	**0.042**	**0.08**	0.052	0.091	0.16	0.68	0.99
3-OHKY	Tryptophan	0.044	0.41	0.42	**0.11**	**0.045**	**0.1**	0.4	0.91
MEL	Tryptophan	**0.49**	**0.044**	**0.08**	0.17	0.5	0.38	0.37	0.91
LD	Tyrosine	0.12	0.19	0.24	0.042	0.67	0.43	0.55	0.92
HVA	Tyrosine	0.1	0.18	0.23	0.021	0.81	0.45	0.47	0.91
VMA	Tyrosine	−0.015	0.79	0.62	−0.0078	0.88	0.46	0.92	1
DOPAC	Tyrosine	−0.014	0.9	0.67	0.12	0.21	0.22	0.37	0.91
MHPG	Tyrosine	−0.21	0.34	0.37	0.11	0.56	0.39	0.27	0.91
TYR	Tyrosine	**0.13**	**0.039**	**0.08**	**0.095**	**0.047**	**0.1**	0.67	0.99
3-OMD	Tyrosine	0.079	0.4	0.42	0.13	0.27	0.26	0.75	1
4-HPAC	Tyrosine	**0.26**	**0.0023**	**0.014**	0.13	0.13	0.17	0.27	0.91
DIOHMAL	Tyrosine	**0.37**	**0.00091**	**0.0074**	0.16	0.054	0.11	0.11	0.91
HGA	Tyrosine	**0.4**	**0.0062**	**0.022**	**0.3**	**0.0099**	**0.058**	0.55	0.92
XAN	Purine	**−0.16**	**0.042**	**0.08**	−0.11	0.086	0.15	0.56	0.92
HX	Purine	−0.0057	0.95	0.69	0.011	0.87	0.46	0.88	1
GR	Purine	0.0002	1	0.7	−0.18	0.51	0.38	0.63	0.99
7-MXAN	Purine	0.083	0.78	0.62	0.051	0.82	0.45	0.93	1
GRMP	Purine	0.052	0.62	0.54	−0.16	0.096	0.16	0.13	0.91
XANTH	Purine	0.047	0.35	0.38	0.0063	0.88	0.46	0.54	0.92
UA	Purine	**−0.058**	**0.056**	**0.095**	−0.015	0.66	0.43	0.34	0.91
METH	One Carbon Meta bolism	**0.2**	**0.0057**	**0.022**	**0.13**	**0.048**	**0.1**	0.48	0.91
4-HPLA	Phenylalanine	**0.13**	**0.00024**	**0.0032**	**0.12**	**8.3E-4**	**0.012**	0.85	1
4-HBAC	Phenylalanine	0.12	0.69	0.59	0.13	0.7	0.45	0.99	1
ATOCO	Antioxidant	**0.63**	**0.0014**	**0.01**	**0.35**	**0.027**	**0.078**	0.24	0.91
DTOCO	Antioxidant	**0.71**	**0.0037**	**0.017**	**0.4**	**0.019**	**0.063**	0.27	0.91
CYS	Cysteine, Glutathione	−0.0014	0.97	0.69	−0.044	0.27	0.26	0.42	0.91
GSH	Cysteine, Glutathione	0.014	0.75	0.62	−0.013	0.74	0.45	0.65	0.99

Changes in absolute concentrations of metabolites (log-transformed) after four week are shown; positive values – up-regulated metabolites, negative values– down-regulated metabolites. Significant changes are shown in bold. See methods section for the details of statistical analysis. Abbreviations: 5-MTPM −5-methoxytryptamine; for the remaining metabolites, see Table 1.

#### Metabolic signatures of placebo after one and four weeks of treatment

Among the patients receiving placebo, a number of metabolites showed significant changes from baseline to the first week of treatment (Table 2); these included metabolites from the purine, tyrosine, and tryptophan pathways, as well as tocopherols. Several metabolites changed significantly from baseline to four weeks in the placebo group (Table 3). These included significant increases in 4-HPLA, HGA, tocopherols, 3-OHKY, TYR, and METH. Compounds changed after one week and four weeks of treatment with placebo are shown within their respective pathways in Figure 1 A–C.

#### Metabolic signatures of sertraline vs. placebo

A comparison of the treatment signatures of sertraline and placebo revealed some interesting patterns (Tables 2 and 3). Many metabolites changes found in the sertraline group were also found in the placebo group, including significant increases in 4-HPLA and HGA after one week of treatment, and increases in 4-HPLA, tocopherols, METH, TYR and HGA after four weeks of treatment. The analysis of differential change yielded a significant effect for 5-HT at one and four weeks. Whereas 5-HT levels decreased in placebo group during the first week, the patients taking sertraline had a greater reduction in serotonin over this time period. Serotonin levels continued to decrease from week 1 to week 4 among the patients taking sertraline. At four weeks 5-HT levels in the placebo group were similar to those at baseline.

Metabolic changes in responders and non-responders are presented in Table S3 and Table S4.

### Associations of Change in Metabolites with Treatment Outcome

We also examined associations between metabolite changes and treatment outcome. Following one week of sertraline treatment, changes in 5-HT and KYN were associated with symptoms reduction from baseline (r = −0.38, p = 0.017 for 5-HT; r = 0.32, p = 0.047 for KYN). For placebo group, changes in DOPAC levels correlated with the treatment response (r = −0.51 and p = 0.00059). A direct comparison of one-week correlations between the two groups, however, did not reveal any significant differences.

After four weeks of treatment, changes in metabolites from tryptophan pathway correlated with the percent change in HAMD_17_ scores in the placebo group, including 5-MTPOL, MEL and 5-HIAA (r = 0.34, p = 0.028; r = 0.32, p = 0.036 and r = −0.31, p = 0.040, respectively). Following four weeks of treatment changes in 4-HPAC had different (p = 0.042) correlations with outcome between sertraline (r = 0.27) and placebo (r = −0.20) groups.

### Analysis of Tryptophan Pathway and Treatment Outcome

**Figure 2:**
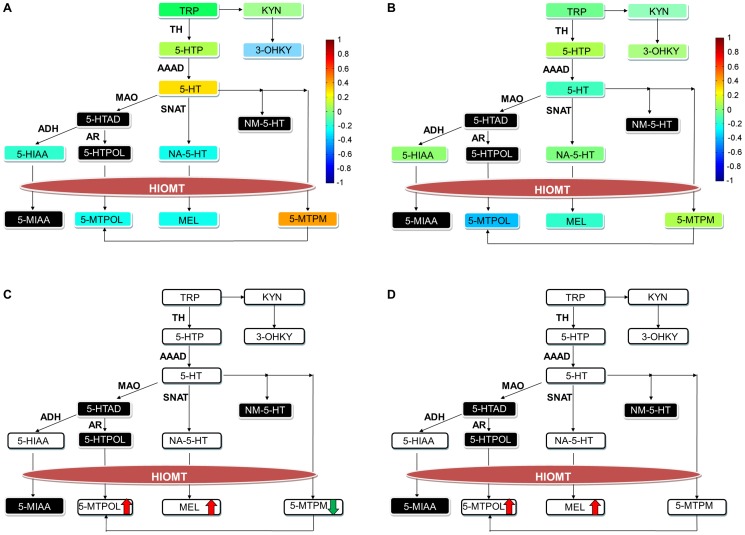
Metabolites within methoxyindole branch of tryptophan pathway correlated with treatment response. Panels A and B show correlations of metabolites at baseline with the four week treatment response in the sertraline and placebo groups, respectively. The correlations are color coded as indicated by the color bar. Panels C and D show four week metabolic changes unique to responders in the sertraline and placebo groups respectively. Abbreviations: HIOMT  =  hydroxyindole-*O*-methyltransferase; 5-HIAD  = 5-Hydroxyindole acetyldehyde; NM-5-HT  =  N-methyl serotonin; 5-HTPOL  = 5-Hydroxytryptophol; 5-MIAA  = 5-Methoxyindole acetic acid; 5-MTPM  = 5-methoxytryptamine. For the remaining metabolites, see Table 1.

Analysis of metabolic changes that correlates with treatment outcome suggests that different branches within tryptophan metabolism might be regulated differently in responders and non-responders to drug and to placebo. Therefore we performed more targeted and comprehensive analysis of the tryptophan pathway, with a focus on the methoxyindole and KYN branches in responders and non-responders to sertraline and placebo (Figure 2). Correlations between baseline levels of individual metabolites, as well as the ratios of individual metabolites within and among branches of tryptophan pathway, and four weeks treatment outcome were evaluated. The ratios between levels of individual metabolites could provide insights about enzymes that are regulated differently in responders and non-responders. The ratios of metabolites across branches of tryptophan metabolism could provide insights about a shift in utilization of substrates for different branches of tryptophan pathway.

### Tryptophan Pathway Analysis and Response to Sertraline

Correlations of pre-treatment metabolite levels and ratios of metabolites to four week percent change in HAMD_17_ scores in the sertraline treatment condition are presented in Figure 2A and Tables 4 and 5. There was a significant positive correlation between pre-treatment 5-MTPM levels and treatment response indicating that higher levels of baseline 5-MTPM were associated with a more favorable 4 week response. None of the other correlations were significant. The ratios of 5-HIAA, NA-5-HT, and MEL to both 5-HT and 5-MTPM tend to be lower in those who responded to treatment. In general, the magnitude of the correlations was greater for the ratios of 5-MTPM to the metabolites of the other branches indicating that responders in the sertraline group are characterized by greater activity in the 5-MTPM branch relative to the other branch of tryptophan pathway.

**Table 4:** Correlations of pre-treatment levels of metabolites within the tryptophan pathway to four-week percent change in HAMD_17_ scores in Sertraline group.

Metabolites	Correlation	p-value	q-value
5-MTPM	0.41	0.013	0.15
5-HT	0.29	0.093	0.35
5-MTPOL	−0.24	0.16	0.35
NA-5-HT	−0.24	0.17	0.35
MEL	−0.22	0.20	0.35
5-HIAA	−0.19	0.28	0.36
5-HTP	0.077	0.66	0.40
TRP	0.051	0.77	0.42

**Note:** A positive correlation coefficient indicates that higher concentrations of metabolites are associated with a better four-week response of depression status.

*Abbreviations*: 5-MTPM  = 5-methoxytryptamine. For the remaining metabolites, see Table 1.

**Table 5:** Correlations of pre-treatment levels of ratios of metabolites within the tryptophan pathway to four week percent change in HAMD**_17_** scores in Sertraline group.

Ratios	Correlation	p-value
5-HTP/TRP	0.072	0.68
5-HT/TRP	0.19	0.28
NA-5-HT/TRP	−0.28	0.10
5-HIAA/TRP	−0.11	0.54
5-MTPOL/TRP	−0.2	0.25
MEL/TRP	−0.26	0.14
5-MTPM/TRP	0.32	0.058
5-HT/5-HTP	0.15	0.40
NA-5-HT/5-HTP	−0.21	0.23
5-HIAA/5-HTP	−0.17	0.34
5-MTPOL/5-HTP	−0.21	0.24
MEL/5-HTP	−0.31	0.068
5-MTPM/5-HTP	0.22	0.19
NA-5-HT/5-HT	−0.33	0.051
5-HIAA/5-HT	−0.33	0.054
5-MTPOL/5-HT	−0.25	0.14
**MEL/5-HT**	**−0.38**	**0.024**
5MTPM/5-HT	0.11	0.53
MEL/NA-5-HT	−0.2	0.26
**MEL/5MTPM**	**−0.4**	**0.016**
**NA-5-HT/5MTPM**	**−0.54**	**0.0007**
**5-HIAA/5MTPM**	**−0.43**	**0.011**
5-MTPOL/5MTPM	−0.28	0.11

**Note:** A positive correlation coefficient indicates that responders favor higher ratios.

*Abbreviations*: 5-MTPM  = 5-methoxytryptamine. For the remaining metabolites, see Table 1.

Binary analysis was used to examine changes in metabolites in responders and non-responders in the four week sertraline group (Figure 2C). There was a significant decrease in 5-MTPM levels in good responders which is consistent with the correlation analysis. At four weeks, responders to sertraline showed significant increases in 5-MTPOL and MEL and a significant decrease in 5-HT. 5-HT levels were also significantly reduced over the four week period in the non-responders; however, 5-MTPOL, MEL, and 5-MTPM levels were not significantly changed in this group. These latter findings suggest that changes in MEL and 5-MTPOL levels could play a role in the mechanisms of response to sertraline.

Analysis of ratios of metabolites between the kynurenine and methoxyindole branches of tryptophan pathway revealed profound differences between responders and non-responders to sertraline (Table 6). Responders to sertraline showed significant decreases in the KYN/MEL and 3-OHKY/MEL ratios from pre- to post-treatment. This suggests that there is a shift in the relative activity from the KYN branch of tryptophan pathway towards melatonin production. The KYN/5-HT ratio also showed a significant increase, but this increase was similar in responders and non-responders. This likely reflects the marked decrease in serotonin seen in both groups.

**Table 6:** Change in tryptophan pathway ratios in responders and nonresponders to sertraline.

Ratio	Responder		Nonresponder	
	Estimate	p-value	Estimate	p-value
KYN/TRP	−0.006	0.75	−0.017	0.40
KYN/5-HTP	0.0057	0.88	−0.076	0.11
KYN/5-HT	0.95	**2.1E-10**	**0.72**	**0.00019**
KYN/NA-5-HT	−0.019	0.48	0.027	0.55
KYN/MEL	−0.28	**0.031**	−0.085	0.59
KYN/5-MTPOL	−0.062	0.090	−0.012	0.86
3-OHKY/TRP	0.013	0.67	−0.04	0.20
3-OHKY/5-HTP	0.025	0.62	−0.099	**0.022**
3-OHKY/5-HT	0.97	**1.2E-09**	**0.69**	**0.00022**
3-OHKY/NA-5-HT	−0.00032	0.99	0.0038	0.94
3-OHKY/MEL	−0.27	**0.040**	−0.11	0.49
3-OHKY/5-MTPOL	−0.043	0.21	−0.035	0.67

*Abbreviations*: For the metabolites, see Table 1.

### Tryptophan Pathway Analysis and Response to Placebo

Similarly to sertraline, we examined associations of tryptophan pathway metabolites and ratios of metabolites to HAMD_17_ changes in the patients treated with placebo. The results of these analyses are summarized in Figure 2B and Tables 7 and 8. There was a significant negative correlation between baseline 5-MTPOL and the change in HAMD_17_ scores indicating that lower pre-treatment 5-MTPOL levels were associated with a better four week outcome. The strongest ratio correlations with outcome were for 5-MTPOL/TRP, 5-MTPM/5-MTPOL, 5-MTPM/5-HT, and 5-MTPOL/5-HTP, although only the first one reached a conventional level of significance.

**Table 7:** Correlations of pre-treatment levels of metabolites within the tryptophan pathway to four-week percent change in HAMD_17_ scores in Placebo group.

Metabolites	Correlation	p-value	q-value
5-MTPOL	−0.40	0.0075	0.49
MEL	−0.16	0.29	0.99
5-HT	−0.15	0.33	0.99
5-HTP	0.093	0.55	0.99
5-MTPM	0.069	0.66	0.99
TRP	−0.044	0.78	0.99
5-HIAA	0.028	0.86	0.99
NA-5-HT	−0.021	0.90	0.99

**Note:** A positive correlation coefficient indicates that higher concentrations of metabolites are associated with a better four-week response of depression status.

*Abbreviations*: 5-MTPM  = 5-methoxytryptamine. For the remaining metabolites, see Table 1.

**Table 8:** Correlations of pre-treatment levels of ratios of metabolites within the tryptophan pathway to four week percent change in HAMD**_17_** scores in Placebo group.

Ratios	Correlation	p-value
5-HTP/TRP	0.14	0.38
5-HT/TRP	−0.15	0.34
NA-5-HT/TRP	−0.044	0.78
5-HIAA/TRP	0.091	0.56
**5-MTPOL/TRP**	**−0.3**	**0.048**
MEL/TRP	−0.1	0.52
5MTPM/TRP	0.039	0.81
5-HT/5-HTP	−0.12	0.45
NA-5-HT/5-HTP	−0.15	0.32
5-HIAA/5-HTP	−0.072	0.65
5-MTPOL/5-HTP	−0.28	0.071
MEL/5-HTP	−0.22	0.16
5MTPM/5-HTP	−0.04	0.80
NA-5-HT/5-HT	0.081	0.61
5-HIAA/5-HT	0.14	0.37
5-MTPOL/5-HT	−0.15	0.32
MEL/5-HT	−0.059	0.71
5MTPM/5-HT	0.29	0.062
MEL/NA-5-HT	−0.16	0.31
MEL/5MTPM	−0.18	0.25
NA-5-HT/5MTPM	−0.00076	1.00
5-HIAA/5MTPM	0.057	0.72
5-MTPOL/5MTPM	−0.29	0.059

**Note:** A positive correlation coefficient indicates that responders favor higher ratios.

*Abbreviations*: 5-MTPM  = 5-methoxytryptamine. For the remaining metabolites, see Table 1.

Analyses of change in responders and non-responders to placebo revealed that there were significant four week increases in 5-MTPOL and MEL in responders to placebo (Figure 2D), a pattern that was similar to that seen in the good responders in the sertraline treatment condition**.** However, 5-MTPM was not significantly changed in good responders to placebo. There were no significant changes observed in the group of poor responders to placebo. There were also significant pre- to post-treatment decreases in the KYN/MEL and 3-OHKY/MEL ratios among good responders to placebo (Table 9). As with sertraline, good responders to placebo show a shift in tryptophan metabolism from KYN production towards the production of MEL.

**Table 9:** Change in tryptophan pathway ratios in responders and nonresponders to placebo.

	Responder		Nonresponder	
Ratio	Estimate	p-value	Estimate	p-value
KYN/TRP	−0.0085	0.67	−0.0027	0.89
KYN/5-HTP	0.043	0.39	−0.0051	0.93
KYN/5-HT	−0.0063	0.89	0.05	0.075
KYN/NA-5-HT	−0.0023	0.95	0.018	0.48
KYN/MEL	−0.37	**0.038**	0.19	0.18
KYN/5-MTPOL	−0.099	0.079	0.038	0.28
3-OHKY/TRP	0.011	0.74	0.034	0.27
3-OHKY/5-HTP	0.063	0.22	0.032	0.60
3-OHKY/5-HT	0.014	0.79	0.086	**0.015**
3-OHKY/NA-5-HT	0.018	0.66	0.055	0.056
3-OHKY/MEL	−0.35	**0.046**	0.22	0.11
3-OHKY/5-MTPOL	−0.079	0.16	0.074	0.075

*Abbreviations*: For the metabolites, see Table 1.

#### Tyrosine Pathway and Treatment Outcome

Correlating pre-treatment metabolite levels to the four-week treatment outcome did not yield any metabolites within the tyrosine pathway that significantly correlate with response to treatment with drug or placebo. An examination of changes in metabolites in responders and non-responders in the sertraline group yielded 4-HPAC and DIOHMAL to be significantly increased in responders (Table S4A). DIOHMAL levels were also significantly increased over the four week period in the non-responders (Table S4B). Significant increases in LD and HGA levels after four weeks were evident in the group showing poor response to sertraline (Table S4B). In the placebo group HGA was increased in non-responders and none of the other metabolites on the tyrosine pathway were significantly changed in this treatment group (Table S4B).

## Discussion

The results of our recent metabolomics study indicated that pretreatment serum metabolic signatures of MDD patients allowed prediction of response to sertraline [30]. The current study extends on these findings by: (1) identifying the biochemical changes that occur in MDD patients one week and four weeks after treatment with sertraline or placebo; (2) identifying which changes are associated with a reduction in depressive symptoms after one and four weeks of treatment with sertraline or placebo.

In the sertraline group, we found a marked decrease in level of 5-HT from baseline to both one and four weeks of the study, which is significantly larger than that in the placebo group. This observation is consistent with previous reports on the effects of SSRIs on blood 5-HT levels and most likely reflects inhibition of 5-HT transporter and decreased levels of 5-HT in the platelets following chronic SSRI treatment [2],[36]. Similar to the previous findings on fluoxetine [2], no correlation between effects of sertraline on blood 5-HT levels and early outcome of the treatment was found. Decreased 5-HT levels were also observed at week one in the patients receiving placebo; whether this early decrease is related to the disease itself or reflects potential effect of placebo needs further investigation with longer observation times and the inclusion of the drug/placebo-naïve patients and healthy control subjects.

In addition to decreased 5-HT and 5-HIAA levels, we observed changes in other metabolites within the tryptophan pathway, both at baseline and after treatment, and these effects were different for responders and non-responders. There was a significant and positive correlation between baseline 5-MTPM levels and treatment response at four weeks, indicating that higher levels of 5-MTPM before treatment were associated with a more favorable response. Levels of 5-MTPM were significantly reduced over the four week treatment period, but only among good responders to sertraline. Additionally, significant differences in the ratios between several metabolites within the tryptophan pathway were observed. The ratios of 5-HIAA, NA-5-HT and MEL to both 5-HT and 5-MTPM were significantly lower in responders to sertraline, compared to non-responders. In the responders to sertraline levels of 5-MTPOL and MEL were significantly increased over the four week treatment period, while in the non-responders levels of 5-MTPOL, MEL and 5-MT were not affected. Neither KYN nor the other metabolites within the kynurenine branch of tryptophan pathway were significantly altered in the group receiving sertraline. Analysis of the ratios showed an increase in KYN/5-HT and 3-OHKY/5-HT ratios following four week treatment both in responders and non-responders to sertraline. The ratios in KYN/MEL and 3-OHKY/MEL were significantly decreased only in the responders. Taken together, these observations suggest that more favorable outcome of the sertraline treatment is associated with the effect on methoxyindole branch of tryptophan pathway, compared to the effect on the kynurenine branch. Increased ratios in KYN/5-HT and 3-OHKY/5-HT might seem to contradict this suggestion. However, levels of KYN and its metabolites, and of methoxyindoles in plasma reflect their respective levels in cerebrospinal fluid [37], while plasma 5-HT levels do not. Therefore, it's more likely that the observed effects on KYN and methoxyindoles are more related to the central effect of sertraline. Several of the changes observed in the sertraline group were also evident in the group receiving placebo, including pre- to post-treatment increases in 4-HPLA, tocopherols, METH, TYR and HGA. The magnitude of these changes was not large and may reflect the progression of the disease or other factors such as changes in behavioral patterns (e.g., changes in diet) related to the recovery process. Perhaps the most important similarities emerged when examining changes in metabolites in responders and non-responders. Similarly to sertraline, significant increases in 5-MTPOL and MEL were found only among responders to placebo. This suggests that response to sertraline and to placebo share some common mechanisms probably reflecting change in depression unrelated to specific treatment that involves methoxyindole branch of the tryptophan pathway. Responders to placebo did not show reduction in 5-MTPM suggesting that decrease in this metabolite be a specific effect that is related to sertraline.

Several studies implicated methoxyindole and kynurenine branches of tryptophan metabolism in the pathogenesis of depression and in mechanisms of action SSRIs [38]–[40]. The results of the present study suggest for the first time that a potential mechanism underlying good or poor response to treatment could be related to the differential effects of the drug on these two branches of the pathway, i.e., engagement of the methoxyindole branch is associated with response to the therapy with SSRI. Analysis of four week change revealed that a greater increase in 5-MTPOL and MEL levels was associated with a greater reduction in HAMD_17_ scores during the four week treatment period. The link between disorganization of circadian rhythms and depression has been well documented, and changed MEL secretion has been observed in the patients with MDD [41]–[43]. Melatonin receptors agonists were proposed as potential novel antidepressants. Efficacy of agomelatine, an agonist of MT1 and MT2 melatonin receptors, in clinical trials was found to be similar to that of SSRIs [44]–[46]. In this respect it is interesting that in animal studies chronic administration of fluoxetine induced several-fold increase in brain arylalkylamine N-acetyltransferase (AANAT) mRNA [47]. AANAT synthesizes NA-5-HT from 5-HT, and NA-5-HT is the first and critical step in MEL synthesis. It was found that NA-5-HT could be not just a MEL precursor but could exert an antidepressant effect by itself, and this effect of NA-5-HT is related to a circadian cycle [48]. These findings, along with the results of our study suggest that heterogeneity within MDD could in part be related to differential regulation of the methoxyindole branch and could underlie the differences in response to therapy with sertraline. The potential role of AANAT and production of melatonin and other methoxyindoles and their contribution to recovery from a depressed state should be explored in greater details.

Although melatonin has been extensively studied as a product of the pineal gland other methoxyindoles are less well studied; both central and peripheral effects have been described for these compounds. 5-MTPOL is regulated during the circadian cycle similarly to melatonin [49],[50] and is involved in regulation of the hormonal axis [51]. 5-MTPM, a tryptamine derivative, closely related to 5-HT and MEL, occurs naturally in the body in low levels and could be synthesized via the deacetylation of melatonin in the pineal gland and contribute to production of 5-MTPOL, and is also involved in the regulation of the hormonal axis [52],[53]. These observations along with the findings of this study suggest that methoxyindoles could be involved in pathogenesis of depression and its treatment outcomes. Circadian rhythm dysfunction is linked to depressive states, [54]–[57], and SSRIs modify circadian locomotor activity rhythms [58]. The relationship between perturbations in the circadian cycle, depression, sleep pattern changes, production of methoxyindoles and regulation of the hormonal axis remains to be further explored.

Limitations of this study include a relatively small sample size and relatively short duration of treatment. Longer treatment periods should help to determine which changes occur earlier, better define the relation between response and biochemical changes, and better discern the ability of early changes to predict latter outcomes. In future studies we will focus on effects of SSRIs and placebo on other pathways, this should extend findings reported and explain variability that exists between responders and non-responders in more details. Additionally correlations between peripheral and central metabolic changes induced by drug and placebo should be investigated. This can lead to the identification of peripheral biomarkers that are disease related and that can be more easily measured. Additionally, a potential limitation of this study is lack of the group of MDD patients without any treatment. While without such group of subjects the possibility still exists that response to placebo could not be distinguished from spontaneous recovery, justification of such clinical trial design could be problematic due to the ethical concerns; future studies are necessary to address this question.

In conclusion, our results suggest a potential role for methoxyindoles in mechanisms of recovery from depressed state, a finding that needs to be followed upon where samples should be collected during the day and night hours to define how the circadian cycle, production of methoxyindoles, changes in sleep patterns and hormonal axis are modified in responders and non-responders to SSRIs and to placebo.

## Supporting Information

Table S1 Subject selection criteria.
(DOCX)

Table S2 Association of demographic variables with percent change in HAMD_17_ score.
(DOCX)

Table S3 Metabolic changes after one week treatment with sertraline and placebo in responders (A) and in non-responders (B).
(DOCX)

Table S4 Metabolic changes after four week treatment with sertraline and placebo in responders (A) and in non-responders (B).
(DOCX)
