# Supplementary material for: Pharmacometabolomics of Response to Sertraline and to Placebo in Major Depressive Disorder – Possible Role for Methoxyindole Pathway
Source: PLoS One. 2013 Jul 17;8(7):e68283. doi: 10.1371/journal.pone.0068283 (PMC3714282; doi:10.1371/journal.pone.0068283)
Supplement: Table S1 — Subject selection criteria. (DOCX) [file pone.0068283.s001.docx]

# Table S1: Subject selection criteria.

| **Inclusion Criteria:** | |
| --- | --- |
| Subjects must meet all of the following inclusion criteria to be eligible for enrollment into the trial: | |
| 1. | Outpatient males or females between 18-65 years of age. |
| 2. | Subjects who are not currently taking psychotropic medications. All subjects must be able to remain free of any prescription or nonprescription psychotropic drugs for two weeks prior to baseline (six weeks in the case of fluoxetine or neuroleptics) until discontinuation from the study. |
| 3. | Subjects must give prior consent to participate in the study and be competent to sign an informed consent. The investigator will be responsible for obtaining written informed consent prior to the participation of a subject in the study. The consent form must be signed and dated by the subject at the time of consent. |
| 4. | Subjects must have a primary diagnosis of MDD by DSM-IV criteria, and symptoms of depression must be present for at least 1 month before screening. |
| 5. | Subjects must have a total score of > 22 on the 17-item Hamilton Depression Scale (HAM-D17) at screening. |
| 6. | Concomitant psychotherapy is permitted, provided there has been no change in the frequency of visits during the three months prior to study initiation, and no change is anticipated during the study. Acceptable therapies include interpersonal, psychodynamic and cognitive behavioral. |
| **Exclusion Criteria:** | |
| Subjects presenting with any of the following will not be included in the trial: | |
| 1. | Subjects who, in the investigator's judgment, would require electroconvulsive therapy (ECT) or treatment with any additional psychotherapeutic drugs including lithium, benzodiazepines, mood stabilizers, and neuroleptics, or would require a change in intensity of psychotherapy during the course of the study. |
| 2. | Known failure to respond (in past 5 years) to two or more trials adequate in duration and dosing of antidepressant therapy. |
| 3. | Known failure to respond to a previous trial of sertraline. |
| 4. | Treatment with neuroleptics or fluoxetine in the 6 weeks before baseline, any other antidepressants within 2 weeks of baseline, any investigational drug within 30 days of baseline, or ECT within 6 months of baseline. |
| 5. | 1. Subjects presenting with any of the following will not be included in the trial:   Any of the following current (within the past 6 months through the present) DSM-IV Axis I diagnoses:   - Generalized Anxiety Disorder, Obsessive compulsive disorder, Panic disorder, Posttraumatic stress disorder, Anorexia, Bulimia, Caffeine-induced anxiety disorder, Alcohol abuse or dependence unless in full remission for at least 1 year - Substance abuse or dependence unless in full remission for at least 1 year as determined by urine drug screen (amphetamines, barbiturates, opiates, benzodiazepines, sedatives and hypnotics, cocaine, phencyclidine (PCP), cannabinoids, or other illegal or illicit drugs) - Social anxiety disorder.   Any of the following past or current DSM-IV Axis I diagnoses:   - Schizophrenia, Psychotic disorder, Delirium, dementia, amnestic, and other clinically significant cognitive disorders, Bipolar or schizoaffective disorder, Cyclothymic disorder, Dissociative disorders. |
| 6. | Subjects judged by the investigator as being at significant risk of suicide or homicide during the study. |
| 7. | Presence of antisocial, borderline or any other personality disorder severe enough in the investigator’s opinion to compromise the subject's ability to comply with the study protocol, including answering self-report questionnaires in a candid fashion. |
| 8. | Subjects who are judged unable or unwilling to follow the protocol. |
| 9. | Any subject with a chronic or life-threatening illness (HIV, cancer, insulin-dependent diabetes, etc) whose medical condition might interfere with an antidepressant treatment response. |
| 10. | Any subject who is likely to be hospitalized (for any reason) during the study. |
| 11. | Subjects with a known hypersensitivity to sertraline. |
| 12. | Subjects requiring treatment with MAOIs, thioridazine, or pimozide. |
| 13. | Subjects with severe, frequent migraine headaches. |
| 14. | Breast-feeding women unless their clinician deems them appropriate for treatment with antidepressant medication. |
| 15. | Women who are currently pregnant, or who have been pregnant within the 6 months prior to joining the study. |
| 16. | Subjects receiving treatment with Type 1C anti-arrhythmics (i.e., flecainide, propafenone). |
| 17. | Subjects whose schedule or activities during the study period will make it difficult to comply with the completion of daily assessments or the scheduling of clinic visits. |
| 18. | Subjects who do not possess a working telephone (land line) due to the potential for being randomly assigned to the electronic diary conditions with the requirement that data be downloaded on a daily basis over the phone line. |
| 19. | Other severe acute or chronic medical or psychiatric condition or laboratory abnormality that may increase the risk associated with trial participation or investigational product administration or may interfere with the interpretation of trial results and, in the judgment of the investigator, would make the subject inappropriate for entry into this trial. |
| 20. | Exclusionary Drugs: |

| **DRUG CLASS** | **Episodic Use** | **Chronic Use** |
| --- | --- | --- |
| Analgesics (Tramadol and products with butalbital are not permitted) | Y | N |
| Antiarrythmics | N | N |
| Antidepressants | N | N |
| Antipsychotics (Thioridazine, Pimozide) | N | N |
| Asthma medications (theophylline is not permitted | N | Y |
| Benzodiazepines | N | N |
| Stimulants (e.g. Strattera, Ritalin) | N | N |
| Oral Contraceptives | N | Y |
| Cough/Cold preparations (diphenhydramine, phenylopropanolamine and dextromethorphan are not permitted) | Y | N |
| Investigational medicines | N | N |
| Mood Stabilizers (Lithium, Divalproex, Carbamazepine) | N | N |
| Monoamine oxidase inhibitors (MAOIs) | N | N |
| NSAIDs (that inhibit both COX 1 and 2) | N | N |
