# Supplementary material for: Pharmacometabolomics of Response to Sertraline and to Placebo in Major Depressive Disorder – Possible Role for Methoxyindole Pathway
Source: PLoS One. 2013 Jul 17;8(7):e68283. doi: 10.1371/journal.pone.0068283 (PMC3714282; doi:10.1371/journal.pone.0068283)
Supplement: Table S2 — Association of demographic variables with percent change in HAMD17 score. (DOCX) [file pone.0068283.s002.docx]

**Table S2:** Association of demographic variables with percent change in HAMD_17_ score.

| **Placebo** | **Race** | White: APC= -41% | Non-White: APC= -59% | P= 0.06 |
| --- | --- | --- | --- | --- |
|  | **Gender** | Male: APC= -42% | Female: APC= -51% | P= 0.33 |
|  | **Age** | PCC = -0.023 | | P= 0.89 |
| **Sertraline** | **Race** | White: APC= - 47% | Non-White: APC= -52% | P= 0.57 |
|  | **Gender** | Male: APC= - 40% | Female: APC= -55% | P= 0.07 |
|  | **Age** | PCC = -0.035 | | P= 0.84 |

Notes: APC=average percent change in HAMD_17_ score; PCC=Pearson’s correlation coefficient.
