# Supplementary material for: Pharmacometabolomics of Response to Sertraline and to Placebo in Major Depressive Disorder – Possible Role for Methoxyindole Pathway
Source: PLoS One. 2013 Jul 17;8(7):e68283. doi: 10.1371/journal.pone.0068283 (PMC3714282; doi:10.1371/journal.pone.0068283)
Supplement: Table S3 — Metabolic changes after one week treatment with sertraline and placebo in responders (A) and in non-responders (B). (DOCX) [file pone.0068283.s003.docx]

**Table S3A:** Metabolic changes after one week treatment with sertraline and placebo in responders.

| **Compound** | **Pathway** | **Sertraline** | | | **Placebo** | | | **Comparison** | |
| --- | --- | --- | --- | --- | --- | --- | --- | --- | --- |
|  |  | **Change** | **p-value** | **q-value** | **Change** | **p-value** | **q-value** | **p-value** | **q-value** |
| TRPOL | Tryptophan | 0.082 | 0.66 | 0.84 | -0.18 | 0.23 | 0.57 | 0.27 | 0.99 |
| 5-HT | Tryptophan | **-0.79** | **2E-6** | **0.00022** | -0.22 | 0.09 | 0.4 | 0.001 | 0.12 |
| 5-HIAA | Tryptophan | -0.14 | 0.076 | 0.36 | -0.1 | 0.14 | 0.46 | 0.74 | 0.99 |
| 5-HTP | Tryptophan | -0.06 | 0.73 | 0.87 | -0.054 | 0.67 | 0.98 | 0.98 | 0.99 |
| 5-MTPOL | Tryptophan | 0.15 | 0.2 | 0.52 | 0.0032 | 0.96 | 0.98 | 0.28 | 0.99 |
| 5-MTPM | Tryptophan | -0.21 | 0.28 | 0.58 | -0.13 | 0.15 | 0.47 | 0.71 | 0.99 |
| KYN | Tryptophan | 0.052 | 0.27 | 0.56 | 0.023 | 0.68 | 0.98 | 0.68 | 0.99 |
| NA-5-HT | Tryptophan | -0.022 | 0.87 | 0.92 | 0.13 | 0.17 | 0.5 | 0.36 | 0.99 |
| TRP | Tryptophan | 0.044 | 0.48 | 0.73 | 0.064 | 0.24 | 0.6 | 0.81 | 0.99 |
| 3-OHKY | Tryptophan | -0.02 | 0.81 | 0.89 | 0.033 | 0.69 | 0.98 | 0.65 | 0.99 |
| MEL | Tryptophan | 0.41 | 0.11 | 0.4 | -0.084 | 0.84 | 0.98 | 0.3 | 0.99 |
| LD | Tyrosine | 0.13 | 0.35 | 0.6 | 0.027 | 0.84 | 0.98 | 0.59 | 0.99 |
| HVA | Tyrosine | 0.13 | 0.31 | 0.59 | -0.014 | 0.92 | 0.98 | 0.42 | 0.99 |
| VMA | Tyrosine | 0.1 | 0.12 | 0.4 | 0.016 | 0.82 | 0.98 | 0.36 | 0.99 |
| DOPAC | Tyrosine | -0.048 | 0.79 | 0.88 | -0.39 | 0.038 | 0.32 | 0.19 | 0.99 |
| MHPG | Tyrosine | -0.33 | 0.21 | 0.53 | -0.17 | 0.68 | 0.98 | 0.74 | 0.99 |
| TYR | Tyrosine | -0.027 | 0.94 | 0.93 | 0.031 | 0.91 | 0.98 | 0.9 | 0.99 |
| 3-OMD | Tyrosine | -0.18 | 0.31 | 0.59 | 0.032 | 0.76 | 0.98 | 0.3 | 0.99 |
| 4-HPAC | Tyrosine | 0.47 | 0.012 | 0.21 | 0.13 | 0.29 | 0.63 | 0.11 | 0.99 |
| DIOHMAL | Tyrosine | 0.2 | 0.26 | 0.56 | 0.24 | 0.15 | 0.46 | 0.88 | 0.99 |
| HGA | Tyrosine | 0.19 | 0.083 | 0.36 | **0.26** | **0.0025** | **0.091** | 0.61 | 0.99 |
| XAN | Purine | -0.21 | 0.045 | 0.29 | **-0.25** | **0.003** | **0.091** | 0.73 | 0.99 |
| HX | Purine | -0.075 | 0.43 | 0.69 | -0.17 | 0.057 | 0.37 | 0.47 | 0.99 |
| GR | Purine | 0.17 | 0.62 | 0.81 | -0.19 | 0.55 | 0.85 | 0.44 | 0.99 |
| 7-MXAN | Purine | -0.51 | 0.31 | 0.59 | -0.38 | 0.45 | 0.78 | 0.85 | 0.99 |
| GRMP | Purine | -0.0083 | 0.96 | 0.93 | 0.011 | 0.93 | 0.98 | 0.92 | 0.99 |
| XANTH | Purine | 0.033 | 0.72 | 0.87 | -0.004 | 0.96 | 0.98 | 0.75 | 0.99 |
| UA | Purine | -0.048 | 0.28 | 0.57 | 0.035 | 0.38 | 0.75 | 0.16 | 0.99 |
| METH | One Carbon Metabolism | 0.0032 | 0.97 | 0.93 | 0.081 | 0.54 | 0.85 | 0.62 | 0.99 |
| 4-HPLA | Phenylalanine | **0.18** | **0.0011** | **0.058** | 0.089 | 0.011 | 0.21 | 0.094 | 0.99 |
| 4-HBAC | Phenylalanine | 0.49 | 0.31 | 0.59 | -0.29 | 0.42 | 0.75 | 0.19 | 0.99 |
| ATOCO | Antioxidant | 0.4 | 0.24 | 0.56 | 0.33 | 0.18 | 0.5 | 0.87 | 0.99 |
| DTOCO | Antioxidant | 0.75 | 0.18 | 0.52 | 0.43 | 0.27 | 0.63 | 0.62 | 0.99 |
| CYS | Cysteine, Glutathione | 0.0024 | 0.96 | 0.93 | 0.033 | 0.53 | 0.84 | 0.68 | 0.99 |
| GSH | Cysteine, Glutathione | -0.02 | 0.41 | 0.68 | -0.023 | 0.4 | 0.75 | 0.93 | 0.99 |

The column of change provides average change of log-transformed concentration after one week of treatment; positive values – up-regulated metabolites, negative values - down-regulated metabolites; significant changes are shown in bold. Abbreviations: 5-MTPM = 5-methoxytryptamine. For the remaining metabolites, see Table 1.

**Table S3B:** Metabolic changes after one week treatment with sertraline and placebo in non-responders.

| **Compound** | **Pathway** | **Sertraline** | | | **Placebo** | | | **Comparison** | |
| --- | --- | --- | --- | --- | --- | --- | --- | --- | --- |
|  |  | **Change** | **p-value** | **q-value** | **Change** | **p-value** | **q-value** | **p-value** | **q-value** |
| TRPOL | Tryptophan | 0.0024 | 0.98 | 0.68 | -0.24 | 0.03 | 0.22 | 0.12 | 0.47 |
| 5-HT | Tryptophan | **-0.72** | **4.3E-07** | **3.4E-5** | -0.2 | 0.086 | **0.31** | **0.0013** | **0.074** |
| 5-HIAA | Tryptophan | -0.19 | 0.0027 | 0.11 | 0.019 | 0.73 | 0.96 | 0.0095 | 0.24 |
| 5-HTP | Tryptophan | -0.14 | 0.15 | 0.36 | 0.0097 | 0.95 | 0.97 | 0.39 | 0.6 |
| 5-MTPOL | Tryptophan | -0.027 | 0.7 | 0.6 | 0.026 | 0.81 | 0.96 | 0.68 | 0.68 |
| 5-MTPM | Tryptophan | -0.052 | 0.58 | 0.59 | -0.13 | 0.2 | 0.49 | 0.55 | 0.62 |
| KYN | Tryptophan | -0.11 | 0.044 | 0.27 | 0.06 | 0.18 | 0.46 | 0.016 | 0.24 |
| NA-5-HT | Tryptophan | 0.027 | 0.68 | 0.6 | 0.0043 | 0.96 | 0.98 | 0.84 | 0.71 |
| TRP | Tryptophan | -0.022 | 0.63 | 0.6 | 0.061 | 0.17 | 0.43 | 0.19 | 0.5 |
| 3-OHKY | Tryptophan | -0.11 | 0.18 | 0.39 | 0.15 | 0.056 | 0.28 | 0.021 | 0.27 |
| MEL | Tryptophan | 0.3 | 0.32 | 0.49 | 0.62 | 0.037 | 0.24 | 0.45 | 0.6 |
| LD | Tyrosine | 0.053 | 0.67 | 0.6 | -0.42 | 0.0085 | 0.14 | 0.017 | 0.24 |
| HVA | Tyrosine | 0.0025 | 0.98 | 0.68 | 0.046 | 0.58 | 0.89 | 0.71 | 0.69 |
| VMA | Tyrosine | 0.12 | 0.11 | 0.36 | 0.061 | 0.39 | 0.76 | 0.56 | 0.62 |
| DOPAC | Tyrosine | -0.18 | 0.16 | 0.36 | 0.41 | 0.0039 | **0.14** | **0.0017** | **0.074** |
| MHPG | Tyrosine | -0.072 | 0.77 | 0.6 | 0.24 | 0.29 | 0.65 | 0.35 | 0.6 |
| TYR | Tyrosine | -0.073 | 0.73 | 0.6 | 0.13 | 0.6 | 0.91 | 0.53 | 0.62 |
| 3-OMD | Tyrosine | -0.082 | 0.35 | 0.5 | 0.23 | 0.17 | 0.43 | 0.097 | 0.46 |
| 4-HPAC | Tyrosine | 0.18 | 0.14 | 0.36 | 0.16 | 0.15 | 0.43 | 0.91 | 0.74 |
| DIOHMAL | Tyrosine | 0.17 | 0.22 | 0.43 | 0.27 | 0.01 | 0.14 | 0.54 | 0.62 |
| HGA | Tyrosine | 0.34 | 0.058 | 0.27 | 0.29 | 0.0035 | 0.14 | 0.78 | 0.69 |
| XAN | Purine | -0.18 | 0.16 | 0.36 | -0.25 | 0.014 | 0.14 | 0.65 | 0.67 |
| HX | Purine | -0.066 | 0.57 | 0.59 | -0.26 | 0.032 | 0.22 | 0.23 | 0.59 |
| GR | Purine | -0.47 | 0.12 | 0.36 | -0.078 | 0.81 | 0.96 | 0.36 | 0.6 |
| 7-MXAN | Purine | 0.16 | 0.66 | 0.6 | 0.13 | 0.67 | 0.95 | 0.95 | 0.74 |
| GRMP | Purine | -0.012 | 0.91 | 0.66 | -0.07 | 0.57 | 0.88 | 0.73 | 0.69 |
| XANTH | Purine | 0.11 | 0.14 | 0.36 | -0.037 | 0.5 | 0.85 | 0.11 | 0.46 |
| UA | Purine | -0.095 | 0.011 | 0.17 | -0.008 | 0.86 | 0.96 | 0.14 | 0.48 |
| METH | One Carbon Metabolism | 0.16 | 0.037 | 0.27 | 0.18 | 0.017 | 0.15 | 0.77 | 0.69 |
| 4-HPLA | Phenylalanine | 0.055 | 0.26 | 0.48 | 0.099 | 0.069 | 0.3 | 0.53 | 0.62 |
| 4-HBAC | Phenylalanine | -0.44 | 0.24 | 0.45 | 0.59 | 0.11 | 0.34 | 0.048 | 0.46 |
| ATOCO | Antioxidant | 0.084 | 0.72 | 0.6 | 0.61 | 0.012 | 0.14 | 0.11 | 0.46 |
| DTOCO | Antioxidant | 0.024 | 0.94 | 0.67 | 0.56 | 0.074 | 0.3 | 0.24 | 0.59 |
| CYS | Cysteine, Glutathione | -0.056 | 0.053 | 0.27 | 0.044 | 0.34 | 0.7 | 0.064 | 0.46 |
| GSH | Cysteine, Glutathione | -0.0084 | 0.71 | 0.6 | 0.0065 | 0.8 | 0.96 | 0.67 | 0.67 |

The column of change provides average change of log-transformed concentration after one week of treatment; positive values – up-regulated metabolites, negative values - down-regulated metabolites; significant changes are shown in bold. Abbreviations: 5-MTPM = 5-methoxytryptamine. For the remaining metabolites, see Table 1.
