# Supplementary material for: Pharmacometabolomics of Response to Sertraline and to Placebo in Major Depressive Disorder – Possible Role for Methoxyindole Pathway
Source: PLoS One. 2013 Jul 17;8(7):e68283. doi: 10.1371/journal.pone.0068283 (PMC3714282; doi:10.1371/journal.pone.0068283)
Supplement: Table S4 — Metabolic changes after four week treatment with sertraline and placebo in responders (A) and in non-responders (B). (DOCX) [file pone.0068283.s004.docx]

**Table S4A:** Metabolic changes after four week treatment with sertraline and placebo in responders.

| **Compound** | **Pathway** | **Sertraline** | | | **Placebo** | | | **Comparison** | |
| --- | --- | --- | --- | --- | --- | --- | --- | --- | --- |
|  |  | **Change** | **p-value** | **q-value** | **Change** | **p-value** | **q-value** | **p-value** | **q-value** |
| TRPOL | Tryptophan | 0.068 | 0.56 | 0.3 | 0.032 | 0.84 | 0.95 | 0.85 | 0.83 |
| 5-HT | Tryptophan | **-2.2** | **5E-10** | **2.3E-08** | 0.04 | 0.73 | 0.95 | **1.8E-11** | **1.8E-09** |
| 5-HIAA | Tryptophan | -0.046 | 0.62 | 0.31 | -0.097 | 0.076 | 0.68 | 0.63 | 0.83 |
| 5-HTP | Tryptophan | 0.014 | 0.89 | 0.35 | -0.074 | 0.52 | 0.85 | 0.56 | 0.8 |
| 5-MTPOL | Tryptophan | **0.17** | **0.047** | **0.056** | 0.25 | 0.039 | 0.68 | 0.56 | 0.8 |
| 5-MTPM | Tryptophan | **-0.42** | **0.0035** | **0.01** | -0.071 | 0.61 | 0.89 | 0.066 | 0.43 |
| KYN | Tryptophan | 0.027 | 0.58 | 0.31 | 0.026 | 0.58 | 0.89 | 0.98 | 0.87 |
| NA-5-HT | Tryptophan | 0.071 | 0.18 | 0.14 | 0.031 | 0.77 | 0.95 | 0.73 | 0.83 |
| TRP | Tryptophan | 0.041 | 0.4 | 0.27 | 0.045 | 0.3 | 0.81 | 0.94 | 0.86 |
| 3-OHKY | Tryptophan | 0.071 | 0.39 | 0.27 | 0.072 | 0.34 | 0.81 | 0.99 | 0.87 |
| MEL | Tryptophan | **0.68** | **0.03** | **0.049** | 0.87 | 0.035 | 0.68 | 0.7 | 0.83 |
| LD | Tyrosine | -0.069 | 0.58 | 0.31 | -0.024 | 0.87 | 0.95 | 0.82 | 0.83 |
| HVA | Tyrosine | 0.12 | 0.19 | 0.15 | -0.029 | 0.76 | 0.95 | 0.26 | 0.74 |
| VMA | Tyrosine | -0.077 | 0.26 | 0.19 | 0.005 | 0.94 | 0.96 | 0.38 | 0.74 |
| DOPAC | Tyrosine | 0.058 | 0.73 | 0.34 | -0.017 | 0.91 | 0.95 | 0.74 | 0.83 |
| MHPG | Tyrosine | -0.16 | 0.55 | 0.3 | 0.081 | 0.78 | 0.95 | 0.54 | 0.8 |
| TYR | Tyrosine | 0.14 | 0.56 | 0.3 | 0.34 | 0.14 | 0.78 | 0.55 | 0.8 |
| 3-OMD | Tyrosine | 0.19 | 0.22 | 0.17 | 0.15 | 0.43 | 0.85 | 0.87 | 0.83 |
| 4-HPAC | Tyrosine | **0.37** | **0.0015** | **0.0098** | 0.015 | 0.89 | 0.95 | 0.019 | 0.38 |
| DIOHMAL | Tyrosine | **0.47** | **0.01** | **0.03** | 0.072 | 0.59 | 0.89 | 0.067 | 0.43 |
| HGA | Tyrosine | 0.21 | 0.31 | 0.22 | 0.2 | 0.32 | 0.81 | 0.98 | 0.87 |
| XAN | Purine | -0.14 | 0.17 | 0.14 | -0.088 | 0.31 | 0.81 | 0.67 | 0.83 |
| HX | Purine | -0.022 | 0.81 | 0.35 | 0.00052 | 1 | 0.98 | 0.86 | 0.83 |
| GR | Purine | -0.26 | 0.45 | 0.29 | -0.039 | 0.93 | 0.96 | 0.68 | 0.83 |
| 7-MXAN | Purine | -0.32 | 0.29 | 0.21 | 0.22 | 0.56 | 0.88 | 0.26 | 0.74 |
| GRMP | Purine | 0.23 | 0.13 | 0.12 | -0.29 | 0.073 | 0.68 | 0.018 | 0.38 |
| XANTH | Purine | 0.0046 | 0.94 | 0.37 | -0.051 | 0.47 | 0.85 | 0.54 | 0.8 |
| UA | Purine | -0.027 | 0.46 | 0.29 | -0.044 | 0.4 | 0.84 | 0.79 | 0.83 |
| METH | One Carbon Metabolism | 0.12 | 0.13 | 0.12 | 0.018 | 0.8 | 0.95 | 0.33 | 0.74 |
| 4-HPLA | Phenylalanine | **0.14** | **0.0043** | **0.023** | 0.13 | 0.022 | 0.68 | 0.86 | 0.83 |
| 4-HBAC | Phenylalanine | 0.07 | 0.89 | 0.35 | 0.6 | 0.31 | 0.81 | 0.49 | 0.8 |
| ATOCO | Antioxidant | **0.76** | **0.0063** | **0.023** | 0.38 | 0.063 | 0.68 | 0.24 | 0.73 |
| DTOCO | Antioxidant | **0.88** | **0.021** | **0.039** | 0.23 | 0.36 | 0.81 | 0.14 | 0.58 |
| CYS | Cysteine, Glutathione | 0.0094 | 0.84 | 0.35 | -0.096 | 0.11 | 0.75 | 0.16 | 0.58 |
| GSH | Cysteine, Glutathione | **0.096** | **0.057** | **0.061** | 0.0066 | 0.91 | 0.95 | 0.23 | 0.73 |

The column of change provides average change of log-transformed concentration after four week of treatment; positive values – up-regulated metabolites, negative values - down-regulated metabolites; significant changes are shown in bold. Abbreviations: 5-MTPM = 5-methoxytryptamine. For the remaining metabolites, see Table 1.

**Table S4B:** Metabolic changes after four week treatment with sertraline and placebo in non-responders.

| **Compound** | **Pathway** | **Sertraline** | | | **Placebo** | | | **Comparison** | |
| --- | --- | --- | --- | --- | --- | --- | --- | --- | --- |
|  |  | **Change** | **p-value** | **q-value** | **Change** | **p-value** | **q-value** | **p-value** | **q-value** |
| TRPOL | Tryptophan | 0.053 | 0.72 | 0.27 | -0.052 | 0.7 | 0.73 | 0.6 | 1 |
| 5-HT | Tryptophan | **-1.6** | **0.00032** | **0.011** | -0.064 | 0.38 | 0.59 | **0.00046** | **0.054** |
| 5-HIAA | Tryptophan | -0.077 | 0.31 | 0.22 | 0.1 | 0.26 | 0.47 | 0.12 | 1 |
| 5-HTP | Tryptophan | **0.24** | **0.023** | **0.069** | 0.063 | 0.58 | 0.68 | 0.23 | 1 |
| 5-MTPOL | Tryptophan | 0.093 | 0.52 | 0.25 | -0.036 | 0.64 | 0.69 | 0.43 | 1 |
| 5-MTPM | Tryptophan | -0.11 | 0.55 | 0.25 | -0.047 | 0.55 | 0.67 | 0.76 | 1 |
| KYN | Tryptophan | 0.066 | 0.11 | 0.12 | 0.051 | 0.33 | 0.56 | 0.82 | 1 |
| NA-5-HT | Tryptophan | 0.0044 | 0.97 | 0.31 | 0.0096 | 0.89 | 0.75 | 0.97 | 1 |
| TRP | Tryptophan | **0.11** | **0.035** | **0.073** | 0.057 | 0.19 | 0.45 | 0.45 | 1 |
| 3-OHKY | Tryptophan | 0.013 | 0.85 | 0.29 | 0.14 | 0.078 | 0.27 | 0.23 | 1 |
| MEL | Tryptophan | 0.26 | 0.5 | 0.25 | -0.38 | 0.25 | 0.47 | 0.21 | 1 |
| LD | Tyrosine | **0.35** | **0.0091** | **0.049** | 0.094 | 0.48 | 0.64 | 0.16 | 1 |
| HVA | Tyrosine | 0.085 | 0.53 | 0.25 | 0.061 | 0.65 | 0.69 | 0.9 | 1 |
| VMA | Tyrosine | 0.058 | 0.55 | 0.25 | -0.018 | 0.81 | 0.75 | 0.53 | 1 |
| DOPAC | Tyrosine | -0.099 | 0.54 | 0.25 | 0.23 | 0.057 | 0.26 | 0.1 | 1 |
| MHPG | Tyrosine | -0.28 | 0.48 | 0.25 | 0.12 | 0.6 | 0.69 | 0.38 | 1 |
| TYR | Tyrosine | -0.11 | 0.72 | 0.27 | 0.25 | 0.25 | 0.47 | 0.33 | 1 |
| 3-OMD | Tyrosine | -0.048 | 0.66 | 0.26 | 0.11 | 0.47 | 0.64 | 0.4 | 1 |
| 4-HPAC | Tyrosine | 0.14 | 0.28 | 0.22 | 0.23 | 0.088 | 0.27 | 0.63 | 1 |
| DIOHMAL | Tyrosine | **0.25** | **0.032** | **0.072** | 0.23 | 0.031 | 0.18 | 0.88 | 1 |
| HGA | Tyrosine | **0.63** | **0.0023** | **0.017** | 0.37 | **0.006** | **0.092** | 0.23 | 1 |
| XAN | Purine | -0.19 | 0.15 | 0.16 | -0.12 | 0.18 | 0.43 | 0.66 | 1 |
| HX | Purine | 0.014 | 0.93 | 0.3 | 0.019 | 0.85 | 0.75 | 0.98 | 1 |
| GR | Purine | 0.31 | 0.43 | 0.25 | -0.29 | 0.42 | 0.61 | 0.26 | 1 |
| 7-MXAN | Purine | 0.56 | 0.32 | 0.23 | -0.084 | 0.77 | 0.74 | 0.31 | 1 |
| GRMP | Purine | -0.16 | 0.25 | 0.21 | -0.057 | 0.63 | 0.69 | 0.57 | 1 |
| XANTH | Purine | 0.098 | 0.27 | 0.21 | 0.052 | 0.34 | 0.56 | 0.65 | 1 |
| UA | Purine | -0.095 | 0.062 | 0.1 | 0.0078 | 0.86 | 0.75 | 0.13 | 1 |
| METH | One Carbon Metabolism | **0.3** | **0.022** | **0.069** | 0.23 | 0.037 | 0.19 | 0.64 | 1 |
| 4-HPLA | Phenylalanine | **0.11** | **0.028** | **0.069** | 0.11 | 0.018 | 0.14 | 1 | 1 |
| 4-HBAC | Phenylalanine | 0.19 | 0.62 | 0.25 | -0.24 | 0.56 | 0.67 | 0.44 | 1 |
| ATOCO | Antioxidant | 0.47 | 0.099 | 0.12 | 0.32 | 0.17 | 0.43 | 0.68 | 1 |
| DTOCO | Antioxidant | 0.52 | 0.091 | 0.11 | 0.53 | 0.024 | 0.17 | 0.98 | 1 |
| CYS | Cysteine, Glutathione | -0.014 | 0.79 | 0.28 | -0.0024 | 0.96 | 0.77 | 0.88 | 1 |
| GSH | Cysteine, Glutathione | -0.084 | 0.25 | 0.21 | -0.028 | 0.6 | 0.69 | 0.52 | 1 |

The column of change provides average change of log-transformed concentration after four week of treatment; positive values – up-regulated metabolites, negative values - down-regulated metabolites; significant changes are shown in bold. Abbreviations: 5-MTPM = 5-methoxytryptamine. For the remaining metabolites, see Table 1.
